# First household survey on drug abuse in São Paulo, Brazil, 1999: principal findings

**DOI:** 10.1590/S1516-31802003000600003

**Published:** 2003-11-06

**Authors:** José Carlos Fernandes Galduróz, Ana Regina Noto, Solange Aparecida Nappo, Elisaldo Luiz de Araújo Carlini

**Keywords:** Marijuana abuse, Illegal drugs, Alcoholism, Epidemiological research design, Household survey, Drogas ilícitas, Alcoolismo, Abuso de maconha, Projetos de pesquisa epidemiológica

## Abstract

**CONTEXT:**

In order to establish prevention programs regarding psychotropic drug use that are adapted to specific populations it is, first of all, important to have data on the realities of such consumption. Single data points are not enough for drawing up a profile of society in relation to drugs.

**OBJECTIVE:**

The aim of this household survey was to determine the incidence of illegal drug, alcohol, tobacco and psychotropic medication use, and thus the number of persons dependent on drugs, alcohol and nicotine, and to evaluate their perception regarding how easy it is to obtain psychotropic drugs.

**TYPE OF STUDY:**

Epidemiological survey.

**SETTING:**

All of the 24 cities in the State of Sao Paulo with more 200,000 inhabitants participated in the study.

**METHOD:**

The sampling was constructed from weighted probabilistic stratified conglomerates obtained via two-stage selection. In each municipality sampled, census sectors (generally 200-300 households) were first selected. Then, households and a respondent were selected to provide information from his/her point of view. The SAMHSA questionnaire (Substance Abuse and Mental Health Services Administration) of the U.S. Department of Public Health was used, after translation and adaptation to Brazilian conditions.

**RESULTS:**

A total of 2,411 persons aged 12-65 years old were interviewed, of whom 39.9% weremen. Lifetime use of any psychotropic drug other than alcohol and tobacco was 11.6%: much less than in the U.S. (34.8%). The alcohol dependence rate was 6%, similar to findings from other countries. Marijuana was the illegal drug most cited as used daily (6.6%): a prevalence much lower than in the U.S. (32.0%). Inhalant use was next in frequency of use (2.7%): about 10 times less than in the United Kingdom (20%). Cocaine use (2.1%) was about 5 times less than in the U.S. (10.6%). There was no report of heroin use, although there was a surprisingly high perception regarding the ease of obtaining heroin: 38.3% said it was easy to obtain.

**CONCLUSION:**

This study supports the implementation of better prevention programs regarding drug abuse in São Paulo state.

## RESUMO

**CONTEXTO:**

Para se estabelecerem programas preventivos sobre o abuso de drogas numa determinada população é necessário antes fazer um diagnóstico da situação por meio de estudos epidemiológicos.

**OBJETIVO:**

Este estudo buscou detectar, na população geral, a prevalência do uso de drogas ilegais, de álcool e tabaco e de medicamentos de psicotrópicos, podendo-se estimar o número de pessoas dependentes em álcool e nicotina e avaliar a percepção delas em relação à facilidade de obter drogas psicotrópicas.

**LOCAL:**

Todas as 24 cidades do Estado de São Paulo com mais de 200 mil habitantes.

**MÉTODO:**

A amostra foi estratificada em conglomerados, probabilística, obtida por duas fases de seleção. Em cada município pesquisado, foram sorteados setores censitários (geralmente consistindo em 200 a 300 residências) na primeira fase, em seguida as casas e os respondentes. O questionário usado foi o SAMHSA (Abuso de Substância e Administração de Serviços de Saúde Mental) do Departamento Norte-americano de Serviço de Saúde Pública, que foi traduzido e adaptado para as condições brasileiras.

**RESULTADOS:**

Um total de 2.411 pessoas foi entrevistado, das quais 39,9% eram homens e todos os participantes estavam na faixa de idade de 12 a 65 anos. Uso na vida de qualquer droga, além de álcool e tabaco, foi de 11,6%, muito menos que nos Estados Unidos (34,8%). Em relação às estimativas de dependentes de álcool, a porcentagem foi de aproximadamente 6%, uma porcentagem semelhante ao observado em estudos de outros países. O uso na vida de maconha foi de 6,6%, muito abaixo que nos Estados Unidos (32%). O uso na vida de inalantes, de 2,7%, foi cerca de 10 vezes menor que no Reino Unido (20%). O uso na vida de cocaína (2,1%) foi aproximadamente cinco vezes menor que nos Estados Unidos (10,6%). Não houve nenhum relato de uso de heroína, embora a percepção da população em relação à facilidade de obter a heroína foi surpreendentemente alta: 38,3%.

**CONCLUSÃO:**

Este estudo apresenta subsídios para a implementação de programas de prevenção adequados à situação de abuso de droga no estado de São Paulo.

## INTRODUCTION

In order to establish prevention programs regarding the use of psychotropic drugs that are adapted to specific populations it is, first of all, important to have data on the realities of such consumption. Single data points are not enough for drawing up a profile of society in relation to drugs.^1^

Basically, three kinds of information are needed for diagnosing the use of psychotropic drugs within a predetermined geographical area:

- General and specific population surveys;
- Statistical indicators;
- Ethnographic surveys.

In relation to the first of these, it can be stated that general population surveys are richest in information on the overall consumption of drugs. These general surveys should obviously be complemented by more segmented surveys, such as those carried out among street children, students, etc.^2^

Other important information on drugs stems from statistical indicators that supply direct data regarding the consequences of their use. Such information includes hospitalization due to dependence, outpatient treatment, admission to emergency rooms and data from Coroners’ Offices. From the latter, forensic reports confirming the use of various drugs can be analyzed. Another statistical indicator that should be noted is the number of drug seizures made by the policing bodies (Federal, Civil and Military Police).^3^

Finally, ethnographic surveys provide qualitative data on the use of a specific drug. In this way, it is possible to determine the specific characteristics of such users.^2^

Even though a considerable amount of information on the use of psychotropic drugs in Brazil is already available, it is essential to have more extensive and up-to-date information so that effective prevention programs can be established.

## OBJECTIVES

The primary objective was to assess the prevalence of lifetime use of illicit drugs, alcohol and tobacco, and also psychotropic medications. The secondary objectives were to estimate the number of people dependent on drugs, alcohol and tobacco, and to understand the perceptions among the population regarding how easy it is to obtain drugs, drug trafficking and the serious risks from using certain drugs.

## METHODOLOGY

### Survey Procedures

The sample design adopted was three-stage sampling via aggregates. In the first stage, census sectors were selected. Next, homes within these sectors were selected. Finally, one respondent in each home was selected. The survey was devised in order to gather household information by means of a sample of self-weighted probabilistic stratified conglomerates.

All the 24 cities in the State of São Paulo with over 200,000 inhabitants participated in the survey. In alphabetical order, these cities are: Bauru, Campinas, Carapicuíba, Diadema, Franca, Guarujá, Guarulhos, Itaquaquecetuba, Jundiaí, Limeira, Mauá, Mogi das Cruzes, Osasco, Piracicaba, Ribeirão Preto, Santo André, Santos, São Bernardo do Campo, São José do Rio Preto, São José dos Campos, São Paulo (Capital), São Vicente, Sorocaba and Taubaté. The total population of these cities is 19,389,903 inhabitants.

The census sectors (usually comprising about 200 to 300 households) are the smallest units for which the IBGE (Brazilian Institute of Geography and Statistics),^4^ provides socioeconomic information. Such information includes the average income of heads of households, number of heads of households with higher education, number of households according to type, etc. These data were used via multivariate statistical techniques for defining groups of homogeneous sectors called strata, in each of the cities selected. The reason for working with such stratified sampling in this type of research is the possibility of increasing the accuracy of the estimates, when using reduced sample sizes. The selection of homes in the census sectors was also carried out by means of the IBGE information.

The number of households surveyed established *a priori* for each sector was 24. The selection of households was made systematically, in which the first household was randomly chosen. This brought the sample closer to a simple random sample. The selection interval for each sector was equal to the total number of homes in the sector divided by 24.

The selection of the respondent in each household was randomly performed, based on a mechanism that did not depend on the interviewer.^5^ The age group selected was between 12 and 65, and only people in that range participated in the selection process.

The interviewers were instructed to start the counting of the homes randomly, in any of the streets belonging to the census sector in question, paying attention to the selection interval previously established. All the interviewers were informed that the counting should not include businesses, hospitals, factories, guesthouses, hotels, etc. If there were properties with apartments, each of the apartments would be equivalent to a home. In general, the interview took place on the second or third visit, because the selected individual was not always present.

The interviewers received training to standardize the procedures for approaching the previously selected residences, in addition to specific training on handling the questionnaire and understanding its content. They were instructed to conduct the interview with the person selected in the most isolated place possible, thereby ensuring freedom and privacy. The purpose was to enhance the credibility of the answers. There were 22 researchers. Each field interviewer was duly identified by a badge with the emblem of the Universidade Federal de São Paulo and carried the questionnaire and a letter inviting the resident to participate in the research. In that letter, the purpose of the research was stated and a guarantee was given regarding the anonymity and confidentiality of responses. It was made clear in this letter that the person selected would only be interviewed if he or she consented to this. In the event of a refusal, the selected person could not under any circumstances be substituted. The study followed all the ethical guidelines of the Declaration of Helsinki.

#### The questionnaire

The questionnaire used was that of SAMHSA (Substance Abuse and Mental Health Services Administration) of the U.S. Department of Health and Human Services Public Health Service, which was translated and adapted to Brazilian conditions.^6^

Basically, the questionnaire consists of seven parts. The first refers to the sociodemographic data of the interviewee. The ABIPEME (Brazilian Association of Market Research Institutes) scale was utilized for classifying the interviewee according to social class (ABIPEME, 1978),^7^ where class A has the highest purchasing power and class E the lowest. The second part of the questionnaire screened for lifetime use of the various psychotropic drugs, and also included anabolic steroids. In the case of a positive response for a certain drug, the interviewer was to delve deeper with questioning on the use of that particular drug during the past year, the month preceding the survey and frequent use (6 times or more in the last month). The latter thus constituted the third part of the questionnaire, i.e. details on each of the drugs. The other parts of the questionnaire will form the subject of a further publication.

To adapt the questionnaire to Brazilian peculiarities, fifty persons answered it twice with an interval of 30 days. Concordance between the first and second interviews was analyzed by means of the Kappa coefficient, utilized for nominal variables.^8^ Overall, the mean Kappa value was 0.79, with extremes of for gender and education and 0.50 for lifetime opiate use.^9^

### Estimates of dependence on alcohol and other drugs

The Diagnostic and Statistical Manual of Mental Disorders, Third Edition Revised (DSM – III – R)^10^ was conceived for use by clinicians and researchers in the diagnosis of psychiatric disorders. The NHSDA (National Household Survey on Drug Abuse) has developed a method for estimating dependence by comparing their own estimates to those of the National Comorbidity Survey (NCS), carried out in 1991.^11^ Those studies concluded that there was significant closeness between the definitions of the DSM – III – R and the NCS. Hence, the NHSDA concept of dependence was adopted in this work.

### Data critique and expansion

The data was scrutinized to uncover inconsistencies in the filling-out of the questionnaire by the interviewer as well as in the responses furnished by the interviewee. The computer program devised for the home survey allowed the detection of these inconsistencies, which were examined one by one. Delphi 5.0 and Sql Server 7.0 were utilized. The most appropriate decision was then taken for each case, resulting possibly in the discarding of the question or even the questionnaire.

The variables regarding the prevalence of psychotropic drug use are considered to be proportions. These can be used for calculating the use of a particular drug in a population, thus obtaining an estimate for the entire population studied. By using this variation coefficient, a description can be obtained of the extent to which the estimate may be affected by sampling errors.^12^

## RESULTS

### Refusal to be interviewed

The refusal rate among the selected individuals was only 2.5%. The largest difficulties in obtaining the interview were among the higher socioeconomic classes and the opposite was seen among the less favored classes.

### Filling-out of the questionnaire

Since this was a questionnaire filled out by an interviewer trained for this purpose, inconsistencies did not exceed 2.0%.

### General characteristics of the sample

The Table 1 shows the distribution of the 2,411 interviewees according to gender and age bracket. It can be seen that there was predominance of male interviewees over female interviewees for the 12-17 and 18-25 year- old age groups. The opposite was observed among those aged over 35 years. Overall, there was a slight predominance of females (53.5%) over males (46.2%).

**Table 1:** Distribution by gender and age group of the 2,411 interviewees living in 24 cities in the State of São Paulo with more than 200,000 inhabitants

	GENDER				TOTAL	
AGE BRACKETS (years)	MALE		FEMALE			
	N	%	N	%	n	%
12 – 17	150	15.6	161	11.1	**311**	**12.9**
18 – 25	177	18.4	230	15.9	**407**	**16.9**
26 – 34	191	19.8	283	19.5	**474**	**19.7**
≥ 35	445	46.2	774	53.5	**1,219**	**50.5**
**TOTAL**	**963**	**100.0**	**1,448**	**100.0**	**2,411**	**100.0**

**Figure 1:**
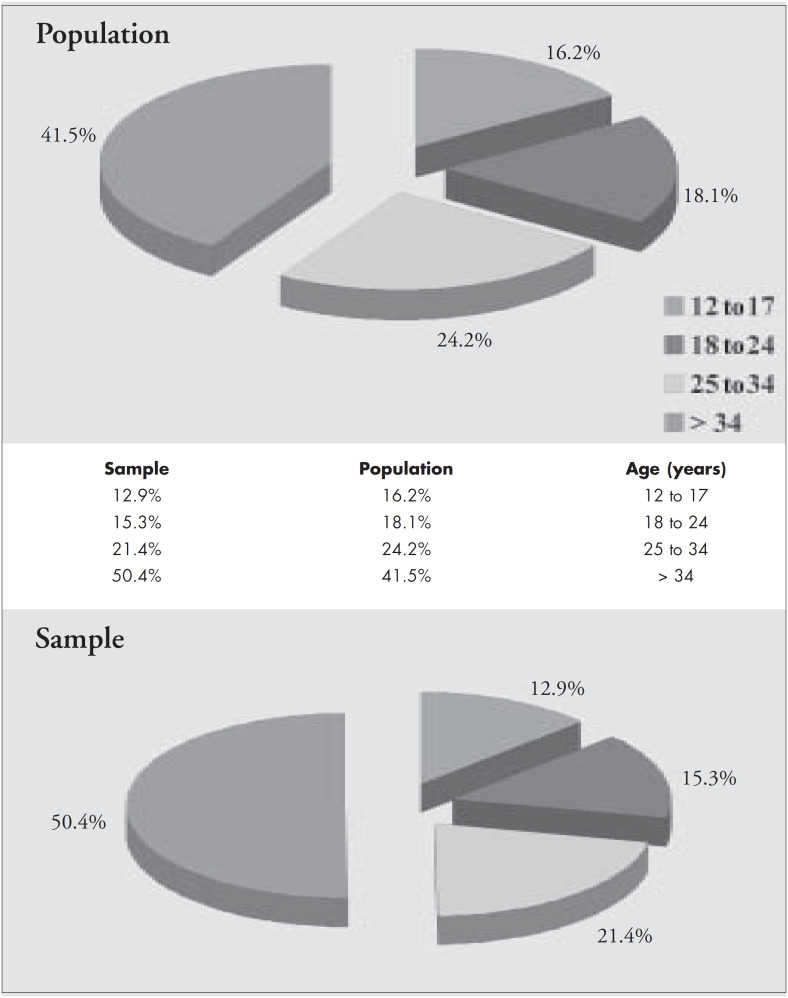
Percentages of the different age groups, comparing the sample obtained and the total population.

Figure 1 shows the data for the age range studied. It can be seen that the percentages for the different age groups are similar when the sample obtained and the total population are compared with IBGE data.^4^

With regard to the schooling of the 2,411 interviewees, about one-third of the sample consisted of illiterates and those who had not finished primary school, independent of gender.

The distribution of the interviewees according to social class was: class A 11.3%; B 31.8%; C 35.6%; D 19.6% and E 1.7%. It can be seen that the socioeconomic classes B and C represented most of the respondents, according to the ABIPEME classification.^7^ This distribution is similar to that of the general Brazilian population.^4^

### Results regarding the use of psychotropic drugs

#### 1. Psychotropic drugs (except alcohol and tobacco)

Table 2 shows the lifetime use of several psychotropic drugs, except tobacco and alcohol, which will be treated separately since they have a different profile of use. Almost 12% of the sample reported the use of some psychotropic drug, corresponding to an estimated drug-using population of 1.72 million inhabitants, not taking into consideration alcohol and tobacco use. Marijuana was the drug most often cited (6.6%) followed by inhalants (2.7%) and cocaine (2.1%). The estimates for the lifetime use of opiate analgesics have low accuracy levels and thus should be interpreted with caution. Nobody reported heroin use (Table 2).

**Table 2:** Prevalence of different psychotropic drugs (except alcohol and tobacco), with lifetime use among the interviewees, in percentages and estimated populations* in the 24 cities in the State of São Paulo with more than 200,000 inhabitants

Drug	%	95% confidence interval
Any Drug	11.6	(1.3 - 12.91)
Marijuana	6.6	(5.6 - 7.6)
Solvents	2.7	(2.0 - 3.3)
Cocaine	2.1	(1.6 - 2.7)
Stimulants	1.2	(0.8 - 1.7)
Benzodiazepine	0.9	(0.5 - 1.2)
Anorectic drugs	0.9	(0.5 - 1.2)
Syrups (codeine)	0.7	(0.4 - 1.0)
Hallucinogens	0.7	(0.4 - 1.1)
Steroids**	0.6	(0.3 - 0.9)
Crack	0.4	(0.2 - 0.7)
Sedatives	0.3	(0.1 - 0.5)
Anticholinergic drugs	0.3	(0.1 - 0.5)
Opiate analgesic	0.2	(0 - 0.3)
Heroin	0	(0 - 0)
**Estimated population**	**(thousands)**	**95% confidence interval**
Any Drug	1,720	(1.533 - 1.907)
Marijuana	979	(834 - 1.123)
Solvents	394	(300 - 488)
Cocaine	318	(233 - 403)
Stimulants	183	(118 - 248)
Benzodiazepine	129	(74 - 184)
Anorectic drugs	126	(72 - 181)
Hallucinogens	110	(59 - 161)
Syrups (codeine)	104	(55 - 153)
Steroids**	96	(49 - 142)
Crack	61	(23 - 99)
Sedatives	49	(15 - 83)
Anticholinergic drugs	49	(15 - 83)
Opiate analgesic	22	(-1 - 46)*
Heroin	0	(0 - 0)

* *Low precision.*

** *Even though anabolic steroids are not considered psychotropic drugs, they are mentioned here due to the rising number of reports on the use of these substances.*

#### 2. Alcohol

Table 3 shows the lifetime use of alcoholic beverages by the interviewees. It can be seen that more males than females used alcohol, in all the age brackets studied. It is worth pointing out that 35.0% of the adolescents (age group from 12 to 17 years) had already made use of alcoholic drinks during their lifetimes.

**Table 3:** Lifetime consumption of alcoholic beverages, divided according to the sex and age group of the 2,411 interviewees from the 24 cities in the State of São Paulo with more than 200,000 inhabitants

Age group (years) and sex	Observed %	95% confidence interval
12 to 17	35.0	(27.4 - 42.6)
Male	37.7	(30.0 - 45.5)
Female	32.3	(25.1 – 39.5)
18 to 24	56.5	(48.9 – 64.0)
Male	66.0	(58.7 – 73.4)
Female	46.9	(40.2 – 53.7)
25 to 34	58.6	(52.1 – 65.1)
Male	67.8	(61.5 – 74.1)
Female	49.7	(44.1 – 55.3)
≥ 35	55.6	(51.1 – 60.0)
Male	70.5	(66.3 – 74.8)
Female	41.5	(38.0 – 45.0)
**TOTAL**	**53.2**	**(51.2 – 55.1)**
**Male**	**63.6**	**(56.2 – 71.1)**
**Female**	**43.0**	**(35.4 – 50.6)**

With regard to estimates of lifetime use of alcohol, the percentage was 53.2%, while the percentage dependent on alcohol was 6.6% (Table 4). In all the studied age groups there were more male dependents than female dependents, reaching almost seven times more among interviewees aged over 35 years (males: 8.9%; females: 1.3%).

**Table 4:** Prevalence of alcohol dependence, divided according to the sex and age group of the 2,411 interviewees from the 24 cities in the State of São Paulo with more than 200,000 inhabitants

Age group (years) and sex	Observed %	95% confidence interval
12 to 17	3.9	(0.8 – 7.0)
Male	5.3	(1.7 – 8.9)
Female	2.5	(0.1 – 4.9)
18 to 24	11.2	(6.4 – 16.0)
Male	18.2	(12.2 – 24.2)
Female	4.3	(1.5 – 7.0)
25 to 34	7.7	(4.2 – 11.3)
Male	12.3	(7.9 – 16.8)
Female	3.3	(1.3 – 5.3)
≥ 35	5.0	(3.0 – 7.0)
Male	8.9	(6.2 – 11.6)
Female	1.3	(0.5 – 2.1)
**TOTAL**	**6.6**	**(5.6 – 7.6)**
**Male**	**10.9**	**(5.9 – 15.8)**
**Female**	**2.5**	**(0.1 – 4.9)**

#### 3. Tobacco

Table 5 shows that 39.0% of the interviewees had used tobacco at least once in their lifetime. Concerning the gender, it can be seen that in all age brackets, males reported predominantly more lifetime use.

**Table 5:** Lifetime use of tobacco, divided according to the sex and age groups of the 2,411 interviewees from the 24 cities in the State of São Paulo with more than 200,000 inhabitants

Age group (years) and sex	Observed %	95% confidence interval
12 to 17	15.8	(10.0 – 21.6)
Male	18.5	(12.3 – 24.7)
Female	13.0	(7.8 – 18.2)
18 to 24	32.7	(25.4 – 40.0)
Male	34.6	(27.2 – 42.0)
Female	30.8	(24.6 – 37.0)
25 to 34	40.4	(33.9 – 47.0)
Male	46.2	(39.5 – 52.9)
Female	34.9	(29.5 – 40.2)
≥ 35	49.9	(45.3 – 54.5)
Male	60.7	(56.2 – 65.3)
Female	39.7	(36.2 – 43.1)
**TOTAL**	**39.0**	**(37.1 – 40.8)**
**Male**	**45.5**	**(37.9 – 53.0)**
**Female**	**32.7**	**(25.6 – 39.8)**

It can be seen that the older the population, the larger the percentage of interviewees reporting lifetime use of tobacco is. Thus, whereas for the 12-17 years of age only 15.8% reported lifetime use, in the group aged over 35 years the percentage exceeded 49%.

In relation to dependence on tobacco, it can be seen that, except in the 18-24 year- old age group, where there are more dependent males, in the other age groups a gender balance is observed in the percentages of tobacco dependence. In total, 10% of the studied population was dependent on tobacco (Table 6).

**Table 6:** Prevalence of tobacco dependence, divided according to the sex and age group of the 2,411 interviewees from the 24 cities in the State of São Paulo with more than 200,000 inhabitants.

Age group (years) and sex	Observed %	95% confidence interval
12 to 17	3.5	*(0.6 – 6.5)*
Male	3.3	*(0.5 – 6.2)*
Female	3.7	*(0.8 – 6.7)*
18 to 24	10.2	*(5.5 – 14.9)*
Male	13.8	*(8.5 – 19.2)*
Female	6.6	*(3.3 – 10.0)*
25 to 34	11.0	*(6.8 – 15.2)*
Male	11.8	*(7.5 – 16.2)*
Female	10.2	*(6.8 – 13.6)*
≥ 35	10.2	*(7.4 – 13.0)*
Male	9.8	*(7.0 – 12.6)*
Female	10.6	*(8.4 – 12.7)*
**TOTAL**	**9.3**	***(8.2 – 10.5)***
**Male**	**10.0**	***(5.2 – 14.7)***
**Female**	**8.7**	***(4.4 – 13.0)***

### How easy it is to obtain drugs, if desired

The interviewees were questioned on the degree of difficulty in obtaining drugs, if they wanted to use them. The following responses were given: 36.2% reported that "it is very easy to obtain LSD (lysergic acid diethylamide)"; 38.3% said that "it is very easy to obtain heroin"; 62.2% said that "it is very easy to obtain cocaine/crack"; 70.2% said that "it is very easy to obtain marijuana" and 83.8% said that "it is very easy to obtain solvents".

On the other hand, the number of people who said they had seen someone selling drugs in their neighborhood in the last 30 days was as high as 18.1%.

### People's opinions about the risks of using some drugs, according to frequency of use

In the following, interviewees’ opinions are presented regarding the risks of using some drugs. Situations of little use and frequent use were put to the respondents. It can be seen that, independent of the drug concerned, the great majority of interviewees considered that regular use represented a serious health risk.

- 48.7% considered that 1 or 2 drinks a week represented a serious risk.
- 95.5% considered that drinking daily represented a serious risk.
- 41.3% considered that using marijuana 1 or 2 times in their lives represented a serious risk.
- 96.2% considered that using marijuana daily represented a serious risk.
- 62.7% considered that using cocaine 1 or 2 times in their lives represented a serious risk.
- 98.9% considered that using cocaine daily represented a serious risk.
- 77.75 considered that using crack 1 or 2 times in their lives represented a serious risk.
- 99.3% considered that using crack daily represented a serious risk.

## DISCUSSION

The first point to be made regarding the survey is the small number of people selected who refused to participate in the interview, amounting to only 2.5% of the total. This percentage reflects the care taken by the researchers in approaching the selected individuals.

The lifetime use of any psychotropic drug, except alcohol and tobacco, was 11.6%, a percentage that is close to that of Chile (17.1%), higher than for Colombia (6.5%) and much lower than for the United States (35.8%).^13-16^ The overall results from the 24 cities surveyed showed that the State of São Paulo has a drug consumption profile that is closer to what is seen for developing countries than to the developed countries of Europe and the United States.

In relation to the lifetime use of any psychotropic drug, according to gender and the age-group, there was a lot of variation in the percentages. Comparison of the results of this household survey with others shows some interesting facts. For instance, in a household study performed in Chile, the lifetime use of any drug (except tobacco and alcohol) in the 12-17 year-old age-group was twice what was seen in the present survey (Chile 10.8%; State of São Paulo 5.1%). When comparing the results from that same age group with data from the USA, it can be seen that the lifetime use in the São Paulo sample corresponds to about a quarter of the American use (22.1%).^13-16^

Moreover, when the lifetime use of drugs among adolescents (12-17 years old) is compared with the Fourth Survey on the Use of Drugs Among Primary and Secondary School Students in the city of São Paulo2, it is observed that the total number of users in the present sample (5.1%) is very much lower than the 19.0% of students that were found to have already tried psychotropic drugs.^2^ This large difference may be due to the way the data were obtained, since the Student Survey was done by means of a self-completion questionnaire, unlike this household survey. As well as this, it is natural that the selected individuals will have had a certain mistrust when in answering a questionnaire on drugs in their own homes. This may in reality reflect discomfort in admitting to the use of illicit drugs, because the percentages for lifetime use of alcohol are very similar to what was found in American research (State of São Paulo 34.7%; USA 38.8%).

Alcohol and tobacco were the drugs with the highest prevalence of lifetime use, accounting for 53.2% and 39.0%, respectively. The lifetime use of alcohol in the 24 major cities of the State of São Paulo was 53.2%. This was lower than the 70.8% observed in Chile and 81.0% in the United States, but higher than what was found in Colombia (35.5%). The size of these differences remained similar for the different age brackets.^14,16,17^

With regard to the estimate of alcohol dependence, this was around 6%, a figure that is close to what has been observed in studies in other countries. It is worth noting that men were almost five times more dependent on alcohol than women (10.9% versus 2.5%, respectively). This is an important aspect to be taken in consideration in preventive programs dealing with alcohol use.

Tobacco was found in the present survey to have been used during the lifetimes of 32.7% females and 45.5% of males. These percentages were lower than the prevalence observed in Chile (70.1%) and the United States (70.5%), and more than double what was seen in Colombia (18.5%).^14,16,17^ The estimates for tobacco dependence were around 10% and were similar for men and women (10.0% and 8.7%, respectively). It is curious to note that, at the two extremities of the studied age groups, women presented greater dependence than men. Even considering that these differences are modest, the data may be reflecting future trends relating to tobacco use and therefore serve as a warning.

Among the illicit drugs, marijuana was the one that presented the largest lifetime use (6.6%). However, the percentages were much lower than those observed in Chile (16.6%), the USA (32.0%), Denmark (31.3%), Spain (22.2%) and the United Kingdom (22.0%).^16-18^

The prevalence of lifetime use of cocaine (2.1%) in the State of São Paulo is very close to that of some countries in South America, such as Chile (2.5%) and Colombia (1.6%), in addition to Holland (2.4%) and Denmark (2.0%). It is also much lower than the prevalence in the United States (10.6%). This suggests that establishing prevention programs copied from other countries without taking Brazilian realities into account is bound to fail.^16-18^

There was no reported use of heroin, which is contrary to what the media has been publishing lately.^19^

The perception of the population of how easy it is to obtain certain drugs was surprisingly high: 38.3% of the interviewees believed it was easy to obtain heroin, 62.4% cocaine and 36.2% LSD. These significant percentages must be part of people's imaginations, since the epidemiological data do not confirm high levels of consumption of these drugs. The perception of drug trafficking presented high percentages, since about 20% stated that they had seen someone selling or trying to buy drugs.

Practically the whole population considered that frequent use of any of the four drugs surveyed in this respect (alcohol, marijuana, cocaine and crack) represented a serious risk. It would seem to be common sense that the use of drugs is harmful to health when this is done abusively. Pointing this out in prevention programs may be worthwhile. The main concern regarding this is that when just the risks of drug use are shown in the prevention campaigns, there is the risk of failure through the attempt at scaring the possible user. Although drug use is known to be harmful, it is important to point out that dependence is an illness and such users have difficulty in abandoning their use. A policy of fear may just trigger anxiety in the user.

The aim of epidemiological research is to ascertain the true picture of a given phenomenon. When it comes to drug consumption, apprehension about declaring such behavior, which is colored by preconceptions and fear, may result in underestimation of the population, depending upon the segment of the population screened, and this should be taken into consideration. Even so, this study supports the implementation of better prevention programs in relation to the drug abuse situation in the State of São Paulo.

## FINAL CONSIDERATIONS

1. The lifetime use of any psychotropic drug, except for alcohol and tobacco, was 11.6%, close to the figure for Chile, greater than for Colombia and much lower than for the USA (34.8%). The overall results from the 24 cities surveyed showed that the State of São Paulo has a profile closer to what is seen in developing countries rather than to the developed countries of Europe or the United States, in relation to the use of psychotropic drugs.
2. Alcohol and tobacco were the drugs with greatest lifetime use, with 53.2% and 39.0%, respectively. The estimates of alcohol dependence were around 6%, close to the figures observed in studies in other countries. Nonetheless, it is worth remembering that these drugs are legalized and prevention campaigns rarely deal with them.
3. Marijuana was the illicit drug that had the greatest lifetime use, although with percentages much lower than observed, for instance, in Chile, United States, Denmark, Spain and United Kingdom. Use among males is greater than among females, a fact that should be taken into account in prevention programs.
4. The prevalence of lifetime cocaine use in the State of São Paulo is very close to that of some countries in South America like Chile and Colombia, as well as Holland and Denmark. It is much lower than the prevalence in the United States. This suggests that the implementation of preventive programs copied from other countries without knowing Brazilian realities will tend to fail.
5. There was no reported use of heroine, which is contrary to what the media has been transmitting recently.
6. The perception among the population of how easy it is to get certain drugs was surprisingly high: 38.3% of the interviewees believed it was easy to get heroine; 62.4% cocaine and 36.2% LSD. These significant percentages must be part of people's imaginations created by the media, because the epidemiological data do not show high consumption of these drugs.
7. The perception of drug trafficking presented high percentages, since about 20% of those surveyed said they had seen somebody selling drugs. On the other hand, the percentages relating to observed trafficking may reflect an enlarged and distorted view of the phenomenon or might even translate into reality, which would be very preoccupying.
8. Almost all the population considered that daily use of any of the four drugs surveyed (alcohol, marijuana, cocaine and "crack") represented a serious risk.

## CONCLUSION

This study supports the implementation of better programs designed to prevent the use of drugs in São Paulo.
